# What Phonological Overlap Effects Reveal About the Role of Working Memory in Native and Nonnative Language Processing

**DOI:** 10.1177/17470218261449150

**Published:** 2026-05-03

**Authors:** Andromachi Tsoukala, Margreet Vogelzang, Ianthi Tsimpli

**Affiliations:** 1 Institut für Niederlandistik, Carl von Ossietzky Universität Oldenburg, Germany; 2 Department of Theoretical and Applied Linguistics, University of Cambridge, UK; 3 School of Psychology, Newcastle University, Newcastle upon Tyne, UK

**Keywords:** phonological overlap, similarity-based interference, silent reading, working memory, nonnative language processing

## Abstract

The present study sheds light on effects of similarity-based interference due to phonological overlap (PO), as well as working memory (WM), during silent reading in native speakers (NS) and nonnative speakers (NNS). While prior research has mainly focused on syntactic complexity or ambiguity to gain insight into nonnative language processing and the role of WM, the effects of PO have remained poorly understood. Using multiline texts with varying degrees of PO, we examined whether increased amounts of overlap disrupt online reading and offline recall, and whether effects differ across native and nonnative groups or vary as a function of WM capacity. Results revealed that greater PO caused delays during online processing, but without impacting offline recall. Crucially, NS and NNS experienced online interference similarly, and WM modulated these effects in comparable ways across both groups. These results suggest convergence both in overt behaviour and in how underlying cognitive resources are used. Findings are discussed with respect to their implications for theories of native-nonnative language processing differences and possible directions for further research.

## Introduction

The question of *whether* and *how* memory resources support real-time language interpretation has concerned researchers for decades. One of the earliest and most influential accounts in this line of research was that of Baddeley (2000) and Baddeley and Hitch (1974), who formalised the concept of working memory (WM). In their account, WM is conceptualised as a limited capacity, multicomponent memory system. It involves the temporary storage and concurrent manipulation of information necessary for performing complex tasks such as comprehension, learning and reasoning. Applied to language processing, WM thus enables us to maintain and update mental representations of linguistic input for long enough to resolve dependencies, derive structure, understand sentences and follow conversations. Baddeley and colleagues’ model has motivated several empirical investigations as well as further theoretical work over the years in order to better characterise the memory system that supports parsing and sentence comprehension (see Adams et al., 2018; Wen, 2016 for reviews).

In previous empirical research, a common approach to studying the role of WM in supporting linguistic functions has consisted of either exerting WM resources or investigating how language processing is affected in contexts typically associated with increased WM demands. Notable examples of such taxing conditions include structurally complex sentences (e.g. syntactic discontinuities, long-distance dependencies), ambiguous sentences and external memory load. Despite their differences, a commonality in all of these cases is that language interpretation becomes effortful as comprehenders have to keep processing incoming input while also holding active burdensome information in WM (e.g. structurally bound sentential elements that are dislocated from their canonical position or are otherwise distant, alternative sentence interpretations, stimuli external to the linguistic task at hand). Another well-studied phenomenon that is of particular interest for present purposes is *similarity-based interference*, that is, the observation that memory representations of sentential elements which overlap on some level of representation (e.g. syntactic, semantic or phonological) lead to confusion when these representations are active in WM (e.g. Gordon et al., 2006). To illustrate, consider sentences containing words that exhibit phonological overlap (PO) as in “The bronze bars were brought in bags to the bank”. Such sentences create interference, making it harder to differentiate, and hence easier to confuse, individual words in WM due to their sound-based similarity, even when read silently (e.g. Baddeley & Hitch, 1974; McCutchen & Perfetti, 1982; McCutchen et al., 1991).

This evidence is derived from research with native speakers (NS). In principle, comparable effects should be observed in proficient nonnative speakers (NNS). However, there is evidence to suggest that contexts associated with heightened WM demands can cause greater difficulty for NNS, often manifesting in slower reading times and poorer sentence comprehension. The cause behind these NS-NNS differences, the extent to which they are fundamental, as well as the role that WM plays in nonnative language processing, continue to constitute debated topics (for reviews, see Clahsen & Felser, 2006; Cunnings, 2017; Hopp, 2022; Juffs & Harrington, 2011; Reichle et al., 2016). Within this literature, much of the empirical evidence has come from studies focusing on NS-NNS differences in the processing of structurally complex or ambiguous sentences; yet, effects of similarity-based interference have remained underexplored. Specifically, little is known about (a) whether NS and NNS experience interference due to PO to similar extents, (b) whether individual differences in WM modulate PO effects in both NS and NNS groups.

In the present paper, we compare the effects of PO in NS and NNS populations during silent reading. Additionally, we examine whether susceptibility to PO effects is associated with individuals’ WM spans. By addressing both group differences and individual variability within groups, our aim is to gain a comprehensive understanding of the role WM plays in supporting language processing, as well as how this role may differ depending on individuals’ language backgrounds and cognitive capacities.

### Phonological Overlap Effects in Native Speakers

The proposition that elements exhibiting similarity on some dimension interfere with memory recall gained research attention in the 60s. Several studies observed that when sequences of words exhibit PO, recall becomes difficult, with memory for the order of words being impacted more so than item memory (Baddeley, 1966; Conrad & Hull, 1964; Craik, 1968; Wickelgren, 1965). In fact, this so-called “phonological similarity effect” was found to be greater compared to the interference caused by other types of item similarity, such as semantic or graphemic similarity between words (Baddeley, 1966).

These findings provided support for the suggestion that phonological information plays an important role in memory for verbal material, which is consistent with Baddeley and colleagues’ WM account. In their original account (Baddeley & Hitch, 1974), the multicomponent model of WM consisted of the *central executive*, which oversaw two slave systems tasked with the temporary maintenance of visual information, termed the *visuospatial sketchpad*, and verbal information, termed the *phonemic buffer* (alternatively known as the *phonological loop*). Under this account, PO effects arise during reading because similar phonological codes are subvocally rehearsed and maintained in the input store of the phonological loop, causing confusion and impairing immediate serial recall. In fact, this recall decrement is not observed when rehearsal is prevented by irrelevant articulation, a procedure known as articulatory suppression (Baddeley et al., 1984). Beyond the word level, little was known about whether and how PO affects sentence processing and interpretation.

One of the first studies examining PO effects on sentence comprehension was conducted by Baddeley and Hitch (1974). In their seventh experiment, they presented participants with written sentences containing word-final PO (rhyme) as in “Red headed Ned said Ted fed in bed” and controls without PO. Half were grammatically and semantically acceptable, and half were not. Participants’ task was to read them, either silently or aloud, and make an acceptability judgement by pressing a response key. Results revealed that participants took longer to provide a response when sentences contained PO. With acceptability judgements taken as a measure closely linked to comprehension (p. 65), the investigators argued that sentence comprehension is susceptible to disruption caused by PO. In extensions to this work conducted by McCutchen and Perfetti (1982) and McCutchen et al. (1991), they argued that this disruption manifests in longer response times because additional time is needed to resolve confusion before comprehension can proceed and the acceptability of sentences can be verified. Since then, subsequent studies have examined PO effects using different methods and materials. Some of these are shown in Table 1, and examples of stimuli they used are shown in Table 2.

**Table 1.** Phonological Overlap Effects in Studies Using Written Stimuli with Native Speakers.

Studies			Behavioural measures			
			Reading times	Response times	Comprehension accuracy	Recall accuracy
Sentence		Baddeley and Hitch (1974) Experiment 7	No effect	Inhibition		
Acceptability		McCutchen and Perfetti (1982) Experiment 2		Inhibition	Inhibition	
Judgements		McCutchen et al. (1991) Experiment 1		Inhibition	No effect	Inhibition^a^
Reading comprehension studies	Simple sentences /text	Haber and Haber (1982)	Inhibition			
		Ayres (1984)	Inhibition		No effect	
		Keller et al. (2003)	Inhibition^b^		Inhibition	
		Frisson et al. (2014) Experiment 2	Inhibition			
	Complex/ambiguous sentences	Kennison (2004) Experiment 2	Inhibition		No effect	
		Acheson and MacDonald (2011) Experiment 1	Inhibition	Inhibition	Inhibition	
		Kush et al. (2015) Experiment 1	Inhibition		No effect	No effect^a^
		Karimi and Diaz (2021) EEG experiment		No effect	No effect	
Memory Recall		Lea et al. (2008) Experiment 2		Facilitation		No effect
		Atchley and Hare (2013)				Facilitation

*Note.* PO = phonological overlap; EEG = electroencephalography.

a In these studies, the column “Recall Accuracy” refers to external memory load performance (i.e. participants had to remember task-external words/digits while reading sentences with PO).

b In Keller et al. (2003), reading times and response times were measured together in the same trial.

**Table 2.** Written Stimuli with Phonological Overlap Used in Studies with Native Speakers.

Studies			Examples with PO^a^
Sentence		Baddeley and Hitch (1974) Experiment 7	Red headed Ned said Ted fed in bed.
Acceptability		McCutchen and Perfetti (1982) Experiment 2	The bronze bars were brought in bags to the bank.
Judgements		McCutchen et al. (1991) Experiment 1	The sparrows snatched the spiders swiftly off the ceiling.
Reading comprehension studies	Simple sentences/text	Haber and Haber (1982)	Five French friars fanned the fainting flea.
		Ayres (1984)	[None provided]
		Keller et al. (2003)	Several short Swedish sword swallowers shifted some swords swiftly.
		Frisson et al. (2014) Experiment 2	A good supply of grain was the holy grail for the farmer following the poor harvest.
	Complex/ambiguous sentencesw	Kennison (2004) Experiment 2	After Dana and David definitely decided to drive, The Datsun began to show signs of mechanical problems.
		Acheson and MacDonald (2011) Experiment 1	The baker that the banker sought bought the house.
		Kush et al. (2015) Experiment 1	It was the boat that the guy who drank some hot coffee sailed on two sunny days.
		Karimi and Diaz (2021) EEG experiment	Jason laughed at Jacob when he was almost drunk and high.
Memory		Lea et al. (2008) Experiment 2	. . .All along the way-winding road, wary whispers of the old barn. . .
Recall		Atchley and Hare (2013)	They let the grasses keep the gems under the grime, given to the earth.

*Note.* PO = phonological overlap; EEG = electroencephalography.

a Apart from the +/− PO manipulation, many of these studies had other conditions too (e.g., +/− acceptability, ambiguity). Not all stimuli versions are shown. Also, note that in Kush et al. (2015), there was no PO within the sentences, but one critical word therein exhibited PO with a task-external list of words.

Within this body of research, most studies have found inhibitory effects on reaction time measures, such that PO leads to longer reading and response times when answering questions about stimuli’s acceptability or meaning. These effects have been detected when various types of materials are used, such as longer text (Ayres, 1984), simple sentences (Keller et al., 2003) and complex or ambiguous structures (Acheson & MacDonald, 2011; Kennison, 2004). Additionally, although there are some differences between word-initial and word-final PO in terms of the time course of effects in online measures (Bridwell, 2017; Frisson et al., 2014), similar findings of inhibition have been reported by studies using repeated rhymes and alliteration. Moreover, there has been some evidence that as the length of sentences and the amount of PO words increase, greater disruption is observed in some measures, such as response times (McCutchen & Perfetti, 1982). Yet, it does not seem to be the case that multiple words exhibiting PO are necessary to observe disruption. Inhibitory effects on reading times have been detected with as few as two PO words, provided that they are in close proximity within the sentence stimuli (Frisson et al., 2014; Paterson et al., 2009).^1^ It is also possible for disruption to be observed even if there is no PO within the sentence stimuli, but rather a critical word therein exhibits PO with a task-external list of words which are actively maintained in memory during the reading task (Kush et al., 2015).

These findings suggest that PO causes delays. As for whether it also affects comprehension accuracy, the evidence is mixed. For instance, the comprehension of sentences with complex embedded clauses, such as centre-embedded object relative clauses, is worse when PO is present as opposed to when it is absent (Acheson & MacDonald, 2011). However, the presence of PO does not seem to affect how syntactic and referential ambiguities are interpreted (Karimi & Diaz, 2021; Kennison, 2004). As for simple structures, some studies assessing comprehension or acceptability judgements have reported lower accuracy in the presence of PO (Keller et al., 2003; McCutchen & Perfetti, 1982), while others have found no such effects (Ayres, 1984; McCutchen et al., 1991).

Similarly, mixed evidence has been reported regarding the effects of PO on recall accuracy, which is a measure of particular interest for present purposes. There has been a long line of research investigating the effects of PO on the recall of various types of item sequences, such as letters, digits and words. Some of these studies have also looked at sentence recall. For instance, research with children has shown that when words within sentences exhibit PO, they become harder to remember (Jorm et al., 1984; Mann et al., 1980). This is not only because the order of words within sentences may be confused (as per the “phonological similarity effect”) but also because other types of errors may be attested, such as omissions, substitutions and intrusions (see also Alloway & Gathercole, 2005 for related evidence). As for research with adults, some studies have examined how recall is affected when sentence stimuli and material external to the linguistic task exhibit PO. In McCutchen et al. (1991), participants memorised a list of digits which they had to recall after reading sentence stimuli. When the digits and the words within sentences started with the same phoneme, such as the voiceless alveolar fricative /s/ sound in the digit 6 and the word “sparrow”, participants recalled fewer digits irrespective of their order compared to when the materials did not exhibit PO. However, Kush et al. (2015), who used a similar paradigm involving external memory load, did not find a PO-induced decrement on recall accuracy.

In contrast to the above, there are contexts in which PO has facilitatory effects on memory recall. For instance, when rhymed words appear at the end of different sentences in reading span tasks (Chow et al., 2016; Macnamara et al., 2011) or at the end of different verse lines in poetic contexts (Goldman et al., 2006; Johnson & Hayes, 1987; Read et al., 2014), participants exhibit better memory for these words. It could be argued that facilitation is observed because PO words are not in close proximity and/or because they do not appear within the same processing unit (e.g. sentences, verse-lines). Indeed, evidence from previous reading studies suggests that as the distance between PO words increases (more than three intervening words), the less likely it is that inhibitory effects will be observed, as the activation of phonological representations decays quickly (Frisson et al., 2014; Paterson et al., 2009). An exception to this is alliteration; in this case, words that start with the same consonant sound appear in close succession within the same processing unit without causing disruption. In fact, memory-related facilitation has been reported. When presented with stand-alone poetic lines that contain alliteration, participants can successfully distinguish them from paraphrased foils that contain different or no alliterative patterns in a recognition task (Atchley & Hare, 2013). Participants are also faster to recognise words that have appeared in alliterative poetic lines and prose when the cues provided at the recognition phase match the alliterative pattern that they had been exposed to, compared to when they do not (Lea et al., 2008). These findings suggest that alliteration creates memory traces for formal sound patterns that can be quickly reactivated, leading to the successful retrieval of words associated with them.

Taken together, the evidence presented in this section suggests that when sentences contain consecutive words that exhibit PO, these sentences take longer to read and comprehend. As for whether sentence comprehension and recall accuracy are affected, the evidence is more mixed and outcomes vary depending on the materials used, the demands of the task and the contexts examined.

### Phonological Overlap Effects in Nonnative Speakers

In the existing literature, it is widely accepted that phonology is automatically activated during fluent silent reading (for reviews of evidence, see Brysbaert, 2022; Clifton, 2015; Rayner et al., 2012), and increases in reading skill have been linked to greater reliance on phonological representations (Alario et al., 2007; Binder & Borecki, 2008; Booth et al., 1999). Consistent with this, children who are skilled readers have been found to be more susceptible to PO interference effects compared to less skilled readers (Mann et al., 1980; however, see Jorm et al., 1984). This has been attributed to greater reliance on phonological representations for maintenance of sentence information in WM (rehearsal in phonological loop). Similarly, greater PO-induced interference has been found in more skilled adult comprehenders compared to less skilled ones during silent sentence reading (Frisson et al., 2014).

The question of whether similar effects emerge in nonnative language processing has received little attention. To the best of our knowledge, only two previous studies have examined the effects of PO during silent reading with NNS populations. Mori (1995) recruited 16 NS of Japanese who spoke English as a second, nonnative language at an intermediate or advanced level of proficiency. The investigator used a similar acceptability judgement paradigm as in McCutchen et al. (1991) and replicated their findings. Specifically, results revealed an effect of PO in response times, as participants took longer to make a sentence acceptability judgement when PO was present as opposed to absent. Similarly, Pélissier et al. (2023) recruited 48 NS of Norwegian who were highly proficient in English as their second, nonnative language. They used a similar paradigm as in Frisson et al. (2014) and additionally examined whether factors related to English proficiency, namely English reading skills and phonological skills, modulated reading time results. Their findings were in line with those reported in the original study. Moreover, individual differences modulated performance; for instance, participants with better reading skills generally read the sentential material faster but spent more time processing critical words, specifically when these exhibited PO compared to when they did not.

These findings provide important insight, suggesting that PO causes delays when processing in a nonnative language, much like what has been observed with NS. It is worth noting, however, that direct NS-NNS comparisons have not been conducted; hence, one research question (RQ1) that remains unaddressed is whether NS and NNS experience PO effects to similar extents and in similar measures when both online processing and offline comprehension or recall are examined. Additionally, individual differences in experiential and proficiency-related factors (e.g. reading and comprehension skills) have been shown to modulate PO effects in both NS and NNS (Frisson et al., 2014; Pélissier et al., 2023). Yet, the influence of individual differences in cognitive resources has not been examined. Specifically, a second research question (RQ2) that remains to be addressed is whether susceptibility to PO effects is associated with individuals’ WM capacity in NS and NNS groups. In what follows, we discuss previous theoretical work and related empirical evidence that can inform hypotheses regarding these RQs.

### Empirical and Theoretical Work on NS-NNS Differences

Empirical work on NS-NNS differences has often found that contexts associated with heightened WM demands can cause greater difficulties when processing a nonnative language, even at advanced levels of proficiency (see Hopp, 2022 for a recent review). Some studies using ambiguous or complex structures have reported that NS and NNS diverge in terms of processing and interpretative patterns (*qualitative* differences; e.g. Felser & Cunnings, 2012; Marinis et al., 2005), whereas others have observed that the two groups converge (e.g. Cunnings & Fujita, 2023; Fujita & Cunnings, 2022), though differences may be observed in the timing or magnitude of effects (*quantitative* differences; e.g. Cunnings et al., 2017; Tsoukala et al., 2024). For instance, effects in NNS may be delayed in online processing, emerging in different regions or measures, and offline comprehension accuracy may be poorer. Similarly, in studies examining PO effects with NNS (Pélissier et al., 2023), PO-induced delays were observed in online measures, just like in NS speakers. However, in some cases, these effects appeared in later regions (after the PO words) compared to what has been reported with NS speakers (Frisson et al., 2014). Yet, note that direct NS-NNS comparisons have not been conducted.

In terms of theoretical work on these NS-NNS differences, two highly relevant models are resource-deficit accounts (Hopp, 2006; McDonald, 2006) and cue-based retrieval accounts (Cunnings, 2017). Both view native and nonnative processing systems as qualitatively similar, and attribute any quantitative differences to WM-related factors. More specifically, resource-deficit accounts argue that because engaging the nondominant language is more cognitively demanding, this limits the resources available to NNS. Thus, any NS-NNS differences are thought to arise from cognitive capacity-based limitations, particularly in WM (e.g. Hopp, 2014). Consistent with this suggestion, NS have been found to experience difficulties similar to those observed in NNS when processing their native language under cognitively taxing conditions (e.g. making acceptability judgements when material is presented rapidly; Hopp, 2010; López Prego & Gabriele, 2014). In parallel, the processing patterns of NS have been shown to resemble those observed in high-span NNS, suggesting that differences between groups are eliminated when appropriate WM resources are available for processing the nonnative language (Dussias & Piñar, 2010; Havik et al., 2009; see also Indefrey, 2006). Similarly, cue-based retrieval accounts argue that NS-NNS differences arise due to a difficulty with certain WM processes, namely susceptibility to interference during memory retrieval operations in the nonnative language. The core idea of this account is that when NNS attempt to retrieve information from WM, they are more likely to be affected by similar but non-target information, which may lead to slower processing and more error-prone comprehension. Although this account primarily addresses interference caused by sentential elements with similar syntactic properties (e.g. Cunnings & Fujita, 2023; Fujita & Cunnings, 2022), it could be extended to similarity-based interference caused by PO: shared phonological features between words in sentences can interfere with retrieval processes, and this may be particularly disruptive for NNS.

Overall, despite their differences,^2^ both of these accounts would explain any differential PO effects between NS and NNS groups by making reference to WM-related factors. For instance, if greater PO-induced disruption in NNS were to be observed, cue-based accounts would attribute this to increased susceptibility to interference and inefficient retrieval strategies in the nonnative language, whereas resource-deficit accounts would attribute this to strained WM resources.

### Individual Differences: WM in a Native and a Nonnative Language

Finally, individual differences in WM capacity are expected to further modulate PO effects. Generally speaking, there is plenty of evidence to suggest that WM makes an important contribution in both native and nonnative language processing. Meta-analyses suggest moderate to small positive associations between WM and native language reading comprehension (Daneman & Merikle, 1996; Peng et al., 2018), as well as between WM and nonnative language processing, reading and proficiency outcomes (In’nami et al., 2022; Linck et al., 2014; Shin, 2020). Three key findings that have emerged from these studies are the following. First, meta-analyses that have examined effects of language status (NS and NNS) have found no significant differences in the correlation strength between WM and reading outcomes (e.g. correlations of .29 for NS and .30 for NNS in Peng et al., 2018). Second, stronger correlations are found with complex WM tasks, such as reading span tasks, compared to simpler ones, such as word or digit span tasks (Daneman & Merikle, 1996; Linck et al., 2014). Third, stronger correlations are found between WM and nonnative reading comprehension when the task measures WM in the nonnative language, rather than individuals’ first language (In’nami et al., 2022; Linck et al., 2014; Shin, 2020). Taken together, these findings suggest that, at least when appropriate WM tasks are used, WM capacity correlates with various aspects of native and nonnative language processing, including reading outcomes.

Regarding this last point, we wish to briefly clarify that by “appropriate WM tasks” we are not suggesting that there is a particular methodological approach that provides a perfect, absolute or process-pure WM measure. On the contrary, obtaining such a pure WM measure may not be practically feasible. As noted in the literature, tasks such as the reading span are designed to tap WM but also likely involve verbal ability and reading skill more generally, in the same way that the operation span task may reflect mathematical ability, inter alia (Conway et al., 2005; Daneman & Hannon, 2007). Thus, task impurity can help explain why reading span tasks tend to correlate more strongly with reading comprehension compared to simpler tasks such as the digit span, namely because there is greater overlap in content and processing demands. Similarly, this overlap matters when considering the language in which WM is tested. As noted by Linck et al. (2014), to the extent that WM task performance requires use of the nonnative language, the task will be an indicator of both WM abilities and skill in the nonnative language, and therefore will not purely measure WM. Crucially, this means that individuals are likely to score lower on WM tasks administered in the nonnative language compared to ones administered in the native language, but this is not due to limitations in WM capacity per se but rather due to limited automaticity and/or resources for processing the nonnative language (Alptekin & Erçetin, 2010; Reichle et al., 2016). Given that WM performance can differ within the same individual depending on various task parameters (e.g. verbal or nonverbal domain, modality, language of testing, etc.), in the present research, we do not treat WM as an absolute trait, but rather as one that is sensitive to the particularities of measurement, including the content of the task and the demands it imposes. Therefore, in light of the complexities involved in obtaining a pure, abstracted WM measure, in this work we adopt a more pragmatic approach: following recommendations of previous research (Alptekin & Erçetin, 2010), we focus on reading span tasks (administered in the nonnative language in the case of NNS) in order to assess WM as it functions under conditions that resemble those involved in reading in that same language.

Having addressed the above, we now turn to relevant literature that has examined the role of WM in NS and NNS, particularly under cognitively taxing conditions (e.g. ambiguity processing). Findings from this body of work suggest a potentially different role of WM for NNS compared to NS groups. For instance, according to resource-deficit accounts, high-span NNS can resemble the processing patterns observed in NS, either only NS with low WM spans (Havik et al., 2009; Hopp, 2014; Indefrey, 2006) or NS overall regardless of their WM capacity (Dussias & Piñar, 2010). This could be taken to indicate that WM plays a particularly important role in nonnative language processing, because processing in a nonnative language may be more reliant on domain-general cognitive resources, such as WM (e.g. the idea of WM as language aptitude, as in Miyake & Friedman, 1998; see also Wen, 2016); as such, higher WM resources in NNS may help “bridge the gap” with NS groups. Although there is supportive evidence for this assumption, there are also claims to the contrary, with some arguing that the influence of WM in NNS is overstated and that other factors, such as exposure and motivation, can impact nonnative language processing outcomes (Juffs & Harrington, 2011). Given the above, there is value in investigating the role of WM in NS and NNS processing, particularly under cognitively demanding conditions, such as PO-induced interference. Beyond providing novel empirical evidence on this understudied phenomenon, this investigation may also inform broader debates regarding the cognitive mechanisms that support native and nonnative language processing.

Given the above, the overarching aim of the current study was to examine effects of PO and WM during silent reading as well as how these may differ between NS and NNS groups. To that end, we conducted a self-paced reading study in which participants read texts that contained word-initial PO, either increased or reduced. After reading each text, participants answered a recall question.

We hypothesised that contexts with increased PO would cause greater interference than contexts with reduced PO. This was expected to manifest in the form of delays in reading times and response times, in line with previous studies’ findings (see Table 1). As for recall accuracy, we did not form strong hypotheses, given the mixed results previously reported in the literature. Additionally, we hypothesised that interference effects would be greater in the NNS compared to the NS group, as would be predicted by cue-based and resource-deficit accounts (e.g. Cunnings, 2017; Hopp, 2006). Finally, we were interested in testing modulatory effects of WM, and particularly whether high-span NNS patterns, together with NS (either low-span NS or NS overall), as has been suggested by proponents of resource-deficit accounts. Following previous studies’ methods (Dussias & Piñar, 2010; Havik et al., 2009), we administered a reading span task and used the median-split approach to categorise NS and NNS into high- and low-span groups.

## Methods

### Participants

Out of the originally recruited 42 native English speakers, 39 formed the NS group (21 female, mean age = 21.3; *SD* = 2.08). Participant exclusions are detailed in the “Data Analysis” section. All were university students in the United Kingdom, recruited through the Prolific platform (https://www.prolific.com/) and mailing lists at the University of Cambridge.

Forty-six NS of Greek who spoke English as a second, nonnative language formed the NNS group (37 female, mean age = 22.4; *SD* = 2.68). The majority of NNS (92%; *N* = 43) were students or recent graduates of English or Translation studies at a Greek university at the time of testing. The rest (8%; *N* = 3) were university students of other degree programmes in Greece. All were recruited through participant calls sent to Greek universities and posted on social media. To assess participants’ nonnative language ability, we administered the British Council’s English Level Placement Test (https://learnenglish.britishcouncil.org/online-english-level-test), as was adapted in Tsoukala et al. (2024). With a maximum possible score of 25, none of the participants scored below 17 or 68% correct (*M* = 21.2; *SD* = 1.63; range = 17 to 24). Based on the British Council’s automatic classification for the test, all participants were at an intermediate or higher level of ability in English.

All participants provided informed consent, and this study has received ethical approval from the relevant ethics committee at the University of Cambridge.

### Materials

#### Self-Paced Reading

In the self-paced reading experiment, the critical stimuli were 16 poem-like texts which consisted of 5 lines. Each line of these items contained word-initial PO in the form of alliteration, as was operationally defined in Lea et al. (2008, p. 710): “a string of three or four instances of the same [word-initial] consonant sound with no more than one intervening, nonalliterative onset consonant sound”. By manipulating the amount of alliterative words within lines, we created two conditions, namely reduced-PO and increased-PO. An example item showing the two conditions can be found in Table 3. A list of all items can be found in the Supplemental Material (see “Data Statement” section).

**Table 3.** Example of Critical Item in the Sel-Paced Reading Experiment.

**Condition:** reduced-PO	**Condition:** increased-PO
Word-initial consonant sound appearing on third, fifth and seventh syllable on all but the third and fourth lines (in red)	Word-initial consonant sound appearing on third, fifth and seventh syllable on all lines (in red)
At the fun and famous fair in the fully fatty food sadly Marshall found a fly whereas Georgie found a flea in the fully fatty food	At the fun and famous fair in the fully fatty food sadly Fletcher found a fly whereas Finnley found a flea in the fully fatty food
**Recall question about third line** (50% of items): Who was it that found a fly? **Recall question about fourth line** (50% of items): Who was it that found a flea? **Response options**: (a) Marshall/Fletcher, (b) Georgie/Finnley, (c) Other(s)	

*Note.* PO = phonological overlap.

Within these items, all lines consisted of seven syllables. In the increased-PO condition, the same word-initial consonant sound appeared on the third, fifth and seventh syllable of every line. Hence, consistent with the aforementioned definition, there were three instances of the same word-initial consonant sound with no more than one intervening, nonalliterative onset consonant sound per line. However, in the reduced-PO condition, the third syllable of line 3 and line 4 contained unrelated word-initial consonant sounds, hence disrupting the alliterative pattern established by preceding lines, and exhibiting a reduced amount of PO.

We deliberately disrupted the alliterative pattern on line 3 and line 4 in the reduced-PO condition because these two lines contained critical information for answering a subsequent recall question. Specifically, the third syllable on both line 3 and line 4 constituted the onset of a proper name. This proper name corresponded to a main character performing an action that participants could be asked about at the recall stage. As such, in the increased-PO condition, where the alliterative pattern was not disrupted, the proper names exhibited PO with preceding and subsequent words in the texts, whereas in the reduced-PO condition, they did not. The aim of this manipulation was to boost the odds of interference in the increased-PO condition, since in that case, PO affected target words; these could be confused due to their sound-based similarity during encoding and/or during post-processing retrieval at the recall stage.

Following each item, participants answered a multiple-choice recall question of the form “Who did what”. For half the items, the question concerned the main character found on line 3, whereas in the other half it concerned the main character found on line 4. The response options were: third-line character, fourth-line character or “Other(s)” as a fallback option.

The items were modelled on stimuli used in previous studies that had also manipulated phonological similarity between proper names (Baddeley & Hitch, 1974; Karimi & Diaz, 2021; Kennison, 2004; see Table 2). To ensure the effectiveness of our items, we performed the following checks. First, to ensure that the proper names had a similar frequency, we consulted registration data for baby names in England and Wales for 2019 by the Office for National Statistics in the UK (Office for National Statistics, 2020). Only disyllabic names listed therein were considered for the stimuli. The two names used in each item were matched as closely as possible for frequency rank (ranked based on the count of registered babies born and given a specific name). A linear mixed effects model (LMEM) with items as a random effect revealed no significant differences in terms of frequency rank between conditions (increased-PO and reduced-PO) or between name position (first and second name within items; *p*’s >.05). Secondly, to ensure that the proper names would be similar in terms of character count, we ran an LMEM which again revealed no significant differences between conditions and name position (*p*’s >.05). Lastly, following Karimi and Diaz (2021), we tested whether the two names in the increased-PO condition actually sounded more similar compared to the two names in the reduced-PO condition. To that end, we calculated the Levenshtein distance between the pronunciation of the two names using the Carnegie-Mellon Pronouncing Dictionary,^3^ version 0.7b (http://www.speech.cs.cmu.edu/cgi-bin/cmudict) and the *adist* function of R. The mean Levenshtein distance in the increased-PO condition (5.5) was smaller than that of the reduced-PO condition (9.06), suggesting that the phonology of the two names was more similar in the former case. A LMEM indicated that this difference was statistically significant (*b* = −3.56, SE = 0.99, *t* = −3.59, *p* = .002). The script and data for these analyses can be found in the Supplemental Material (see “Data Statement” section).

Alongside the critical items, participants also read 96 five-line texts, inclusive of non-critical items meant for unrelated studies (Tsoukala et al., 2024, 2025), as well as fillers, which were followed by comprehension questions to gauge participants’ engagement and attentive reading throughout the task. The critical items were counterbalanced and equally distributed across two lists. Participants saw eight items per condition, and each item was seen in only one of its versions. List assignment and the order in which all stimuli appeared was fully randomised. The order of appearance of the response options, namely third-line and fourth-line character, was pseudorandomised. In each list, for half the stimuli, the third-line character would appear in the leftmost position of the screen, whereas for the rest it would appear in the middle position of the screen. The option “Other(s)” would always appear in the rightmost position.

#### Reading Span Task

We used a variant of the Daneman and Carpenter (1980) reading span task, as was adapted and administered in Swets et al. (2007). In brief, the task involved participants silently reading sentences for which they had to make acceptability judgements, while also memorising words that appeared below the sentences (see Figure 1). The task was computerised and consisted of 36 sentence-trials, divided into 8 sets. The size of each set varied between 3, 4, 5 and 6 sentence-trials, and there were two sets per set size. In half of the trials, the sentences were plausible and made sense (target response: Correct; key-to-press: F). The sentences, along with the words-to-be-recalled, were visible on the screen for 5 s, and it was during this time that participants had to press a key, while also memorising the word in red appearing below each sentence. At the end of each set, participants had to report back to the experimenter all the words they saw in red in the order in which they saw them. The time allowed for reporting varied depending on the set size (12 s were allowed for a set size of 3, 16 s for a set size of 4, 20 s for a set size of 5 and 24 s for a set size of 6). The guidelines laid out by Swets et al. (2007) were followed for scoring. Points were awarded on a trial-by-trial basis. For a point to be awarded for a trial, the word-to-be-recalled had to be reported in the correct form and mentioned in the order in which it appeared in a set, and participants needed to be accurate in their key press for that trial. The maximum possible score was 36. Both the NS and NNS groups completed this task in English.

**Figure 1.**
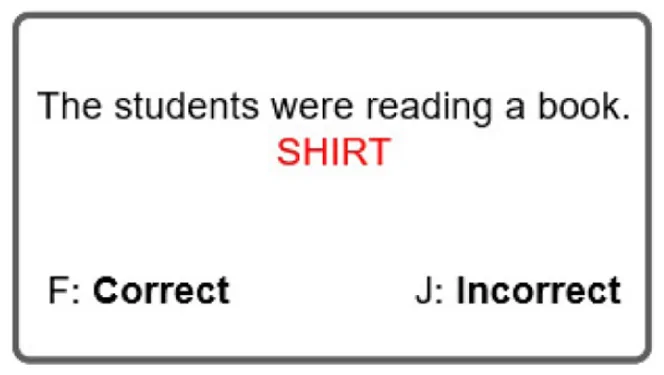
Example of trial in the reading span task.

### Procedure

A web-based reading experiment was designed, which employed the self-paced (line by line) moving-window paradigm (Just et al., 1982). The main reading experiment and the reading span task were programmed in JsPsych (de Leeuw, 2015). To approximate lab-based experimental conditions, a remote testing method was used which involved participants completing the study while being on live call with an experimenter. During testing sessions, participants started the main reading experiment, after going through three practice items. The experiment was split in five blocks. The first 4 blocks contained 22 texts each, while the last one consisted of 24 texts. In between blocks, participants could take a short break and then proceed to complete an additional task. For the NS group, the additional task was the reading span, and for the NNS group, the additional tasks included the reading span and the English Level Placement Test.^4^

We structured the web-based experimental sessions in this way to prevent participant fatigue and loss of interest caused by the long and repetitive nature of the reading experiment. In between breaks, participants would rest and also interact with the experimenter to complete an additional task, thus ensuring continued engagement throughout the session. The order of the tasks was fixed so as to streamline the administration and manual scoring of oral responses that were provided in certain tasks. Had the order of tasks been randomised, there would not have been sufficient preparedness to complete the above steps efficiently.

Within the blocks of the main reading experiment, participants read the texts one line at a time. At the beginning of each trial, only dashes would be visible to mask all the lines. Participants had to press a key to reveal only a single line of text each time, making their way from the first to the fifth one with each key press. After each text, participants answered a question by pressing a key corresponding to one of the response options.

### Data Analysis

Prior to analysis, we checked whether participants had responded accurately to comprehension questions that followed filler items. Following Fernández’s (2002) methodology, participants in the NS group with more than 20% incorrect responses were excluded (*N* = 3), and so were participants in the NNS group with more than 30% incorrect responses (none). As a result, data from 3 individuals in the NS group were discarded, yielding a sample size of 39. Then, trials in which participants had responded with “Other(s)” to the question that followed critical items were excluded (0.5% data loss). Subsequently, reading times for each line and question response times were checked for outliers. Inspection of histograms as well as skewness and kurtosis values indicated that the data were non-normally distributed. We applied a combination of winsorisation^5^ as well as log transformations (Nicklin & Plonsky, 2020), and then rechecked the distribution of the data. The new skewness and kurtosis values suggested that reading times were near normally distributed (range = −0.25 to 0.25), and that response times were slightly positively skewed (range = 0.26 to 0.75; see Blanca et al., 2013 for thresholds). Finally, regarding WM, we used the median-split approach to categorise participants as high span or low span based on their performance in the reading span task. This process yielded a new dichotomous measure which we refer to as “Group Reading Span”.

Analyses were performed in R version 4.4.2 using the lme4 package (Bates et al., 2015). Only reading times for the critical lines that we manipulated (i.e. three and four), as well as question response times, were entered as dependent variables into LMEMs. The responses to the question were entered as a binomial dependent variable into generalised LMEMs. We took the following modelling steps. Firstly, we started with an empty model and used the Akaike Information Criterion to identify the random effects structure that best fitted the data. Provided the model converged, the “maximal” random effects structure included by-participant and by-item intercepts and slopes for Condition; however, often non-convergence issues led to the exclusion of random slopes. Subsequently, Group (negative level: NS), Condition (negative level: reduced-PO) and Group Reading Span (negative level: high span) were deviation-coded and entered in all models as fixed effects along with their interactions. In the analyses of reaction time measures, we also added character count to account for length differences between items. For interactions, we performed post-hoc tests with false discovery rate corrections for multiple comparisons. We report Cohen’s *d* as an index of effect size. The data and scripts for the above are provided in the Supplemental Material (see “Data Statement” section).

## Results

Mean reaction times and accuracy results are shown in Table 4. Reading times are plotted in Figure 2. A summary of the statistical analysis results can be found in Table 5.

**Table 4.** Mean Reaction Times in Milliseconds and Accuracy Results by Group, Condition and Group Reading Span (Standard Error in Parentheses).

Measure	Condition				Difference	
	Reduced-PO		Increased-PO		Increased-PO–reduced-PO	
	High span	Low span	High span	Low span	High span	Low span
NS group						
Line 1 reading times	1,620 (0.06)	1,659 (0.04)	1,543 (0.05)	1,677 (0.04)	−77	18
Line 2 reading times	1,932 (0.05)	1,759 (0.03)	1,750 (0.05)	1,768 (0.04)	−182	9
Line 3 reading times	1,947 (0.04)	1,965 (0.03)	1,884 (0.04)	2,018 (0.03)	−63	53
Line 4 reading times	1,684 (0.04)	1,742 (0.03)	1,791 (0.04)	2,033 (0.03)	107	291
Line 5 reading times	806 (0.04)	1,006 (0.04)	739 (0.03)	1,004 (0.03)	−67	−2
Question response times	2,187 (0.03)	2,414 (0.03)	2,285 (0.03)	2,440 (0.03)	98	26
Recall accuracy	89.6% (2.89)	88.3% (2.95)	90.4% (2.28)	85.8 (2.88)	−0.8	2.5
NNS group						
Line 1 reading times	2,635 (0.04)	3,800 (0.04)	2,629 (0.04)	4,010 (0.04)	−6	210
Line 2 reading times	2,617 (0.03)	3,422 (0.03)	2,642 (0.03)	3,398 (0.03)	25	−24
Line 3 reading times	2,863 (0.03)	3,580 (0.03)	2,771 (0.03)	4,032 (0.03)	−92	452
Line 4 reading times	2,602 (0.03)	3,396 (0.03)	2,830 (0.03)	3,945 (0.03)	228	549
Line 5 reading times	1,068 (0.04)	1,333 (0.04)	1,063 (0.04)	1,265 (0.04)	−5	−68
Question response times	2,925 (0.03)	3,326 (0.03)	2,935 (0.03)	3,514 (0.03)	10	188
Recall accuracy	92.0% (1.75)	88.5% (2.37)	93.2% (2.57)	90.6% (2.16)	−1.2	−2.1

*Note.* The mean log-transformed reaction time data were back-transformed in milliseconds scale. PO = phonological overlap; NS = native speakers; NNS = nonnative speakers.

**Figure 2.**
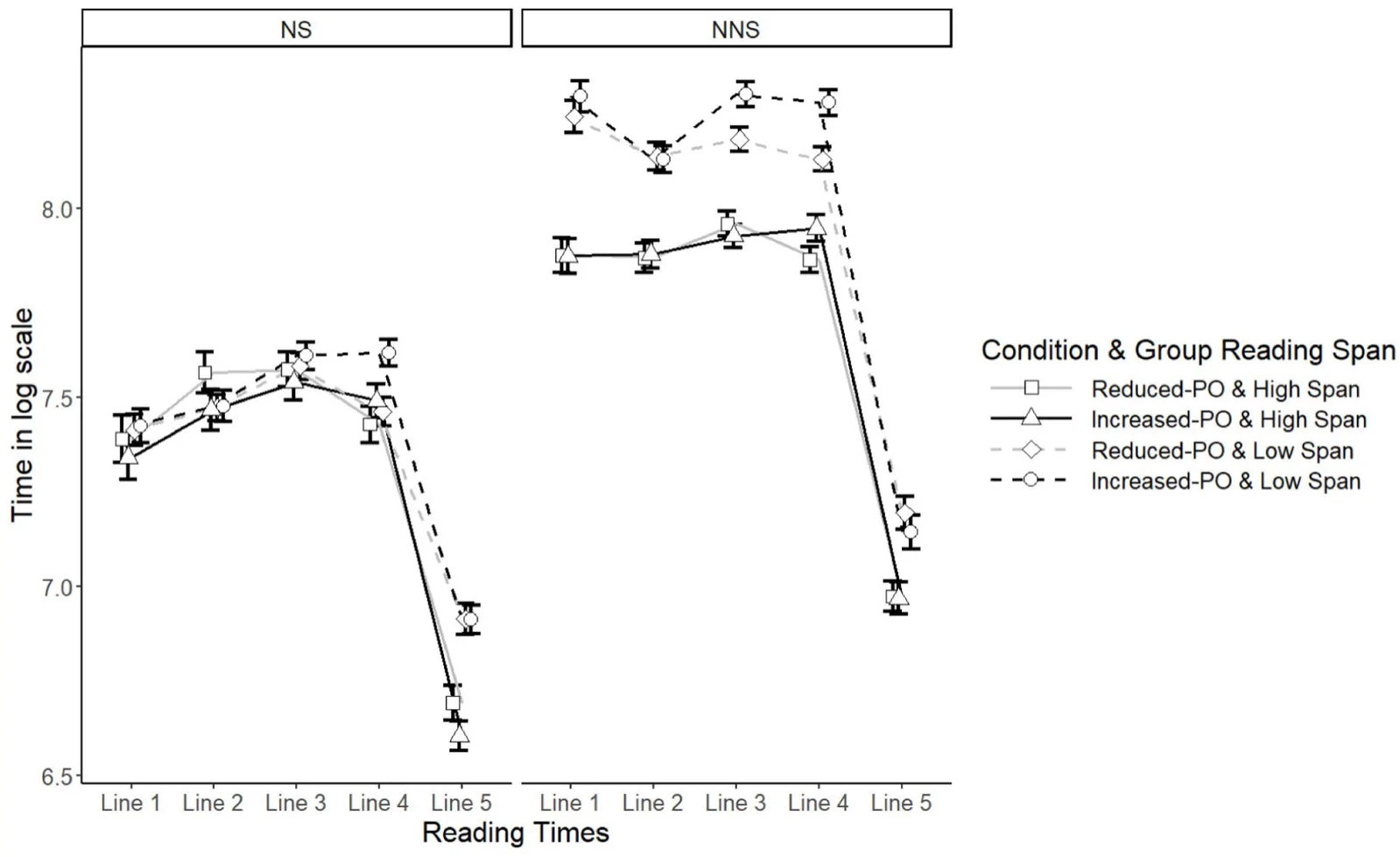
Mean reading times (centered) by Group, Condition and Group Reading Span (SE error bars). *Note.* SE = standard error.

**Table 5.** Summary of Statistical Analysis Results.

Measure	Predictors	Estimate	SE	*z/t*	*p*
Recall accuracy	(Intercept)	2.50	0.21	11.83	**<.001**
	Group	0.29	0.23	1.26	.207
	Condition	0.06	0.18	0.35	.725
	Group reading span	−0.34	0.23	−1.48	.139
	Group × Condition	0.29	0.37	0.78	.433
	Group × Group reading span	−0.11	0.47	−0.24	.809
	Condition × Group reading span	−0.15	0.38	−0.39	.694
	Group × Condition × Group reading span	0.42	0.73	0.58	.562
Line 3 reading times	(Intercept)	7.79	0.18	41.52	**<.001**
	Group	0.51	0.06	8.29	**<.001**
	Condition	0.01	0.02	0.85	.394
	Group reading span	0.16	0.06	2.70	**.008**
	Line 3 characters	0.001	0.008	0.23	.821
	Group × Condition	0.04	0.04	0.96	.335
	Group × Group reading span	0.25	0.12	2.06	**.042**
	Condition × Group reading span	0.09	0.04	2.29	**.021**
	Group × Condition × Group reading span	0.09	0.08	1.17	.242
Line 4 reading times	(Intercept)	6.91	0.26	26.35	**<.001**
	Group	0.55	0.06	8.38	**<.001**
	Condition	0.10	0.02	4.39	**<.001**
	Group reading span	0.18	0.06	2.80	**.006**
	Line 4 characters	0.03	0.01	3.31	**.004**
	Group × Condition	−0.009	0.04	−0.23	.812
	Group × Group reading span	0.21	0.13	1.65	.102
	Condition × Group reading span	0.03	0.04	0.78	.435
	Group × Condition × Group reading span	−0.008	0.08	−0.09	.921
Question response times	(Intercept)	7.82	0.29	26.80	**<.001**
	Group	0.30	0.04	6.84	**<.001**
	Condition	0.02	0.02	1.23	.228
	Group reading span	0.11	0.04	2.61	**.010**
	Question characters	0.003	0.01	0.28	.778
	Group × Condition	0.002	0.04	0.05	.953
	Group × Group reading span	0.07	0.08	0.78	.436
	Condition × Group reading span	0.01	0.04	0.26	.793
	Group × Condition × Group reading span	0.08	0.08	1.05	.292

*Note.* SE = standard error. Significant *p* values (*p* < .05) are highlighted in bold.

All participants’ recall accuracy was above chance levels (mean = 89%; *SD* = 9, range = 56 to 100). Statistical analyses of recall accuracy results revealed no significant effects (*p*’s >.05). Regarding reading times on line 3, a significant effect of Group was detected (*b* = 0.51, *p* < .001; *d* = 1.34, 95% CI [1.01, 1.67]), as NNS exhibited longer reading times. There was also a significant effect of Group Reading Span (*b* = 0.16, *p* = .008; *d* = 0.43 [0.10, 0.76]), as low span readers were slower. The interaction between Group and Group Reading Span was significant (*b* = 0.25, *p* = .042). To follow up on this interaction, we performed post hoc comparisons. Results revealed that all comparisons were significant, expect for one. Specifically, low-span NNS were slower when compared to high-span NNS (*b* = 0.29, *p* = .001; *d* = 0.77 [0.32, 1.21]), low-span NS (*b* = 0.64, *p* < .001; *d* = 1.67 [1.22, 2.12]), and high-span NS (*b* = 0.68, *p* < .001; *d* = 1.78 [1.29, 2.26]). Moreover, high-span NNS were slower when compared to low-span NS (*b* = 0.34, *p* < .001; *d* = 0.90 [0.44, 1.35]), and high-span NS (*b* = 0.38, *p* < .001; *d* = 1.00 [0.52, 1.49]). The difference between low-span NS and high-span NS was not significant (*p* = .669). Finally, there was also a significant interaction between Condition and Group Reading Span (*b* = 0.09, *p* = .021). Post hoc comparisons revealed two key result patterns: (a) in the increased-PO condition, low-span readers were slower than high-span ones (*b* = 0.21, *p* = .0095; *d* = 0.56 [0.21, 0.91]), but there was no such difference in the reduced-PO condition (*p* = .095); (b) in the low-span group, reading times in the increased-PO condition were slower than in the reduced-PO one (*b* = 0.06, *p* = .0405; *d* = 0.17 [0.02, 0.32]), but no such difference was found in the high-span group (*p* = .316).

Regarding line 4 reading times, an effect of Group was detected (*b* = 0.55, *p* < .001; *d* = 1.5, 95% CI [1.13, 1.86]), as the NNS group displayed a slower reading rate. Additionally, there was an effect of Condition (*b* = 0.10, *p* < .001; *d* = 0.27 [0.13, 0.40]), as the increased-PO condition led to longer reading times. Character count also had a positive effect (*b* = 0.03, *p* = .004). Finally, there was an effect of Group Reading Span (*b* = 0.18, *p* = .006; *d* = 0.49 [0.13, 0.86]), as low-span readers were slower.

In terms of question response times, the effect of Group was significant (*b* = 0.30, *p* < .001; *d* = 0.84, 95% CI [0.58, 1.09]), as the NNS group responded more slowly. There was also an effect of Group Reading Span (*b* = 0.11, *p* = .010; *d* = 0.32 [0.07, 0.57]), as the low-span group exhibited a slower response rate. There were no other significant effects in any of the models reported above.

The key results of this analysis can be summarised in the following points. First, we expected that increased PO would cause delays in reaction time measures. We did indeed find such evidence in the reading times of the critical lines that were affected by the PO manipulation. More specifically, compared to contexts with reduced PO, increased PO contexts led to longer reading times on line 3 for low-span individuals only, and to longer reading times on line 4 for all participants. There were no effects in response times or recall accuracy.

Second, we found that the NNS group generally had a slower reading and response rate compared to the NS group. Yet, we did not find that NNS were differentially affected by increased PO (no interactions between Group and Condition). Thus, even though we expected to observe greater disruption in NNS based on previous theoretical work (Cunnings, 2017; Hopp, 2006), the present results do not provide support for this hypothesis. Instead, we found that increased PO caused similar delays in both the NS and NNS group.

Third, we found significant modulatory effects of WM on performance measures. Compared to high-span individuals, low-span individuals exhibited slower reading and response rates. We also found significant interactions of reading span on line 3 reading times. For instance, the interaction between Condition and Group Reading Span suggests that low-span readers were slower than high-span ones only in increased PO contexts; by contrast, low-span and high-span readers did not differ in reduced PO contexts. Additionally, the interaction between Group and Group Reading Span suggests that low-span readers in the NNS group were significantly slower than all other participants, while high-span NNS were also slower than both high- and low-span NS; by contrast, high- and low-span participants in the NS group did not differ. These significant differences between high-span NNS and NS participants, both high- and low-span ones, are not consistent with the suggestion that high-span NNS pattern together with NS groups, as per resource-deficit accounts. We revisit and discuss further these effects, or lack thereof, in the “Discussion” section.

In brief, these findings suggest that increased PO contexts cause delays in online processing for both NS and NNS groups, while also revealing significant modulatory effects of WM. Yet, it is important to note two factors that limit the insight that can be gained from these analyses.

Firstly, the non-significant interaction between Group and Condition does not confirm that NS and NNS participants are equally susceptible to PO-induced interference. In frequentist terms, non-significance indicates only that the data do not provide strong enough evidence to reject the null hypothesis, but it does not quantify support for it. For this reason, we conducted additional analyses in which we computed Bayes factors to quantify the relative evidence for the alternative versus the null hypothesis. Specifically, we compared pairs of models for reading times and response times that differed only in the inclusion versus exclusion of the interaction between Group and Condition. Across these model pairs, we varied (a) model complexity (same model terms as in the main analysis, as well as simpler models without the three-way or multiple two-way interactions); (b) functions used to compute them (Bayes factors were obtained via the BayesFactor R package by Morey et al., 2018, as well as using the brms R package by Bürkner, 2017 with bridge sampling) and priors specified (both weakly informative and more informative priors were specified, closely following relevant prior work by Veríssimo, 2025). Further details regarding these analyses are available via Supplemental Material (see “Data Statement” section). The Bayes factors obtained from these models did not provide strong support for the alternative hypothesis (BF_10_ estimates ranged from 0.04 to 1.02, with most values clustering between 0.07 and 0.14). In other words, for the majority of models, the obtained Bayes factor estimates suggest the data were roughly 7 to 14 times more likely under the models without the interaction between Group and Condition than under the models including it, although several comparisons yielded values closer to 1, suggesting little to no preference between models in those cases. Taken together, these findings suggest a tendency for the data to favour the null hypothesis rather than the alternative hypothesis, though this evidence is not consistent across all model specifications.

Moreover, another complicating factor concerns processing rate differences between participants. For instance, the NNS group exhibited an overall slower reading and response rate than the NS group. This is, in fact, not surprising, as reading in a nonnative language typically takes longer than reading in one’s native language (Brysbaert, 2019). Similarly, as can be seen in Table 4 and Figure 2, low-span NNS exhibited a slower processing rate overall, not just in critical regions. As such, it becomes difficult to discern whether differences between the PO-increased and the PO-reduced conditions (i.e. the magnitude of PO-induced interference) are comparable between subgroups of participants – such as between high-span NNS and low span/all NS, as may be expected based on previous research (Dussias & Piñar, 2010; Havik et al., 2009) – namely because these are overshadowed by baseline reading rate differences.

In order to address this outstanding question, we re-run analyses for reaction time measures, but in this case, we used participant mean-centred logged data. Through centreing, each participant’s own mean per measure is subtracted from their individual trial values, thereby rescaling the data such that each participant has a mean of zero. Essentially, this data transformation allows us to isolate within-participant effects – namely differences between the two conditions and how they may interact with group and group reading span – from between-participant variation in reading/response rate.^6^

Centred reading times are plotted in Figure 3. Analyses revealed only a significant interaction between Condition and Group Reading Span on line 3 (*b* = 0.09, *p* = .018). Post hoc comparisons revealed that the largest difference (*t*.ratio = 2.35) was the one between the two conditions for low-span participants, which was nevertheless rendered non-significant after applying corrections (*b* = 0.67, *p* = .11; *d* = 0.18, 95% CI [0.03, 0.33]). All other differences were smaller and did not reach significance (*p*’s >.05). Regarding line 4 reading times, there was an effect of Condition (*b* = 0.10, *p* < .001; *d* = 0.28 [0.17, 0.38]). Importantly, there were no interactions, suggesting that the magnitude of PO-induced interference was similar for all participants. Finally, there were no significant simple effects or interactions in response times (*p*’s >.05).

**Figure 3.**
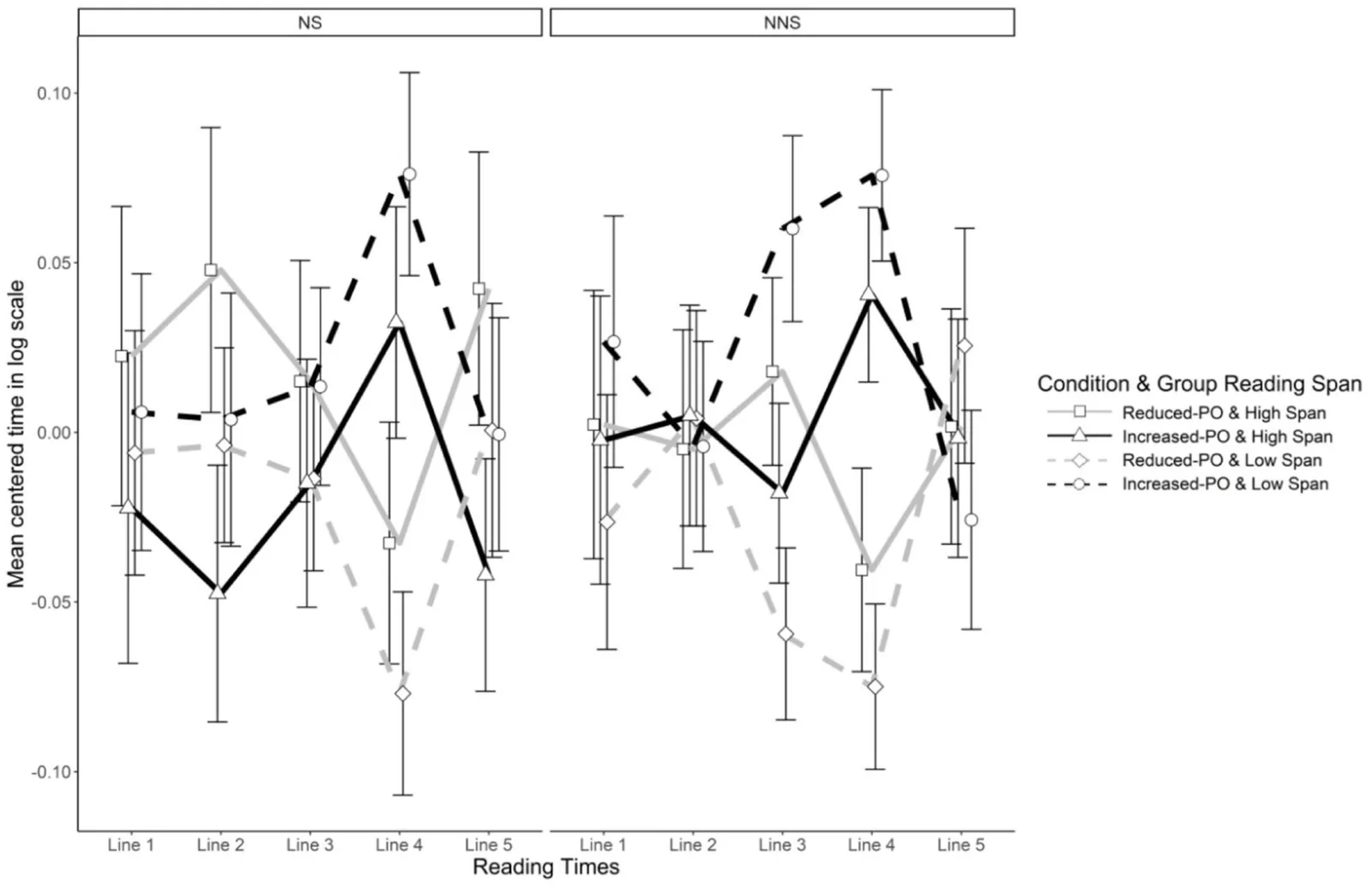
Mean reading times by Group, Condition and Group Reading Span (SE error bars). *Note.* SE = standard error.

Overall, this additional analysis clarified that once baseline processing differences between NS and NNS, as well as between high- and low-span individuals, were factored out, all participants exhibited comparable PO-induced interference. Where interactions did emerge, they did not suggest disproportionate delays for NNS or that high-span NNS in particular patterned together with low span/all NS. Instead, we observed a trend suggesting that low-span individuals in both groups experienced earlier processing costs than high-span participants (i.e. on line 3 in addition to line 4).

## Discussion

The overarching aim of the present study was to shed light on an understudied topic, namely, whether reading in a native and a nonnative language is influenced by similarity-based interference due to PO. The two key research questions we sought to address were: (a) whether NS and NNS experience PO effects to similar extents and in similar measures, and (b) whether susceptibility to PO effects is associated with individuals’ WM capacity in NS and NNS groups.

Regarding the former, our results provide evidence that increased PO induces measurable interference during online processing, consistent with relevant prior research (Acheson & MacDonald, 2011; Ayres, 1984; Frisson et al., 2014; Haber & Haber, 1982; Keller et al., 2003; Kennison, 2004; Kush et al., 2015). Specifically, when two consecutive regions in the texts contained excessive PO, all participants took longer to process the second one of them, suggesting that the build-up of PO caused delays. Interestingly, this online disruption did not extend to post-processing measures: neither response times to recall questions nor accuracy were significantly impacted, although NNS exhibited numerically higher recall accuracy than NS, as can be seen in Table 4. This pattern could reflect a speed-accuracy trade-off, whereby slower processing may have allowed for more attentive consolidation of information, leading to slightly higher accuracy. While it is difficult to speculate whether this could have been a strategic choice or not, the possibility remains that factors other than the ones targeted by our design may have influenced the relationship between online processing costs and offline memory performance. Relating this finding to relevant prior work, it is worth noting that variable outcomes have previously been reported, especially when it comes to recall/recognition accuracy (e.g. no interference effects on accuracy in Kush et al., 2015; Lea et al., 2008). Thus, the presently observed outcomes may reflect this broader variability in the literature and leave open avenues for future research to examine factors such as speed–accuracy trade-offs, strategic processing, and the sensitivity of offline measures to interference effects.

Additionally, results revealed that PO-induced interference was expressed in the same measures and to similar extents in the NS and NNS groups. Although NNS had an overall slower reading rate than NS, as is also commonly observed in the wider literature (Brysbaert, 2019), the magnitude of PO-induced processing delays did not differ between NS and NNS groups. As such, these results do not clearly support resource-deficit or cue-based retrieval accounts, which are expected to predict greater processing costs for NNS under cognitively demanding conditions (Hopp, 2014), particularly in cases involving similarity-based interference (Cunnings, 2017). Nevertheless, it aligns with emerging evidence from studies that have also found no significant NS-NNS differences in the magnitude of interference effects caused by similarity in syntactic representations (Cunnings & Fujita, 2023; Fujita & Cunnings, 2022). To account for these findings, these studies considered that greater interference costs may emerge in NNS only in certain circumstances (e.g. when there are additional or increased task demands) or when larger sample sizes are tested. It has also been suggested that group differences in susceptibility to similarity-based interference may be smaller than previously assumed (i.e. potentially overestimated effects, as discussed in Fujita & Cunnings, 2022). Another possibility is that the proficiency level of the NNS group in this study may have been high enough that their reliance on WM resembled that of NS participants. This may have obscured interaction effects that may have otherwise been observed in NNS groups with lower proficiency levels, consistent with prior theoretical work. We thank an anonymous reviewer for pointing this out.

Moreover, our results do not clearly support the assumption within resource-deficit accounts that high-span NNS pattern together with low-span NS or NS overall. The additional analyses we performed to directly address this question – in the absence of confounding differences in baseline processing rate – did not provide clear evidence for such patterns. Instead, our findings are compatible with a role for WM in modulating PO-induced interference, which we discuss below in relation to our second research question.

Overall, WM capacity affected result patterns both in terms of online reading times and offline response rates, as low-span individuals were slower across the board. Importantly, the effects of PO in online processing varied as a function of reading span. Specifically, low-span individuals exhibited delays in both critical regions affected by the PO manipulation (i.e. line 3 and line 4), suggesting that interference impacted their processing earlier than high-span participants who only exhibited delays in the second critical region (i.e. line 4). This early, low-span-specific delay was significant in initial analyses and non-significant thereafter, indicating a pattern that, while requiring caution, remains worthy of some discussion: for individuals with lower WM capacity, the build-up of similar phonological representations may quickly stretch the resources available for maintaining and integrating information, leading to interference at earlier stages of processing.

Collectively, these findings provide important insight that can also inform broader debates regarding the extent of NS-NNS differences and the role of WM. As noted in the introduction, nonnative language processing has been argued to be particularly vulnerable to similarity-based interference and heavily reliant on WM resources. Yet, in the present study, the effects of PO and the modulating role of WM were not exclusive or more marked in the NNS group. Our results suggest that NS and NNS experienced disruption due to excessive PO to similar extents and in similar processing measures. We also found that WM resources played a comparable role in modulating PO effects across groups. Of note, a trend suggested that low-span individuals became susceptible to interference earlier than high-span participants. These findings indicate that, at least under the circumstances presently examined, native and nonnative language processing converge both in overt processing patterns as well as in the underlying cognitive mechanisms relied upon and the influence they exert. Thus, while NS-NNS differences may still emerge in other circumstances, the current evidence indicates that they are not a deterministic outcome, a finding that could inform theoretical work.

Before concluding, we wish to comment on the strengths and limitations of this study, and offer suggestions for future research directions. Unlike previous research, which has mainly used short sentence stimuli, we employed multiline texts in which each line contained PO. Admittedly, the texts we used exhibited certain stylistic particularities often found in poetry (e.g. short verse-lines, alliteration) which may have influenced result patterns. At the same time, an advantage of this design is that it allowed us to examine how interference unfolds over time during reading, and at what point the build-up of PO is most likely to lead to processing disruption. Future research could focus on better understanding such interference effects in real-time processing, as subtle effects may go undetected in offline measures alone. This was also the case in our study, since we detected effects in online measures, but not in offline ones. One reason for this could be that both of our experimental conditions included PO, albeit to varying degrees. We acknowledge that comparisons involving more stark or categorical differences between conditions (e.g. presence versus absence of PO) may have yielded qualitatively distinct outcomes. That said, our decision to manipulate PO through minimal lexical changes was deliberate, and the finding that such subtle manipulations can yield detectable effects contributes novel insight to the existing literature. Additionally, using this design, we found some evidence to suggest that WM may not only modulate the magnitude of interference, but also its timing: low-span individuals appeared to become susceptible to interference earlier in the text, a pattern that remains to be corroborated and investigated further to determine its reliability and generalizability across contexts and populations. Related to this, it is also important to note that the NNS sample in our study involved highly advanced speakers of English as a nonnative language, with continued text-based exposure to it due to university studies. Thus, it remains to be established whether disproportionate PO effects emerge in NNS groups with different proficiency and exposure levels to the nonnative language. Additionally, our WM assessment in the NNS group was conducted in their nonnative language. While this approach aligns with recommendations of prior research, it also means that the measurement of WM may be influenced by the level of proficiency/automaticity in the nonnative language, rather than reflecting pure WM capacity. Subsequent studies might benefit from including more than one task to assess WM, ideally measuring performance in different domains, including the native and nonnative language. Lastly, this investigation was designed to assess hypotheses of accounts focusing on NS-NNS differences, and as such, engagement with other (psycho)linguistic and memory-related accounts was beyond its scope. Such integration remains an important direction for future work.

## Conclusion

Circling back to points presented in the introduction, there have been ongoing discussions regarding the role of memory resources in supporting real-time language interpretation, and potential differences in this respect between native and nonnative language processing. The present findings contribute to these debates by shedding light on understudied effects of PO and the modulatory role of WM in both NS and NNS groups during silent reading. Our findings demonstrate that greater amounts of PO lead to measurable online interference effects that are further modulated by individuals’ WM capacity. Importantly, all these effects were similarly expressed across the NS and NNS groups. This suggests convergence both in overt reading patterns and in terms of how underlying cognitive resources are used to support processing and manage phonological interference. As such, the assumption that nonnative language processing is more susceptible to similarity-based interference, or that it becomes more challenging under cognitively taxing conditions in general, may need to be reconsidered. Future research can help narrow down the specific conditions under which group differences emerge.

## Data Availability

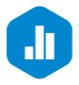 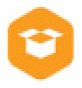 Anonymised data, scripts, and research materials are available at: https://osf.io/uhtav.

## Notes

1. It is worth noting that the purpose of studies included in Tables 1 and 2 varies. For instance, some have examined PO effects on memory encoding and retrieval processes during reading (e.g. Karimi & Diaz, 2021; Kush et al., 2015), while others have focused on lexical access (e.g. Frisson et al., 2014). Despite differences in study focus, a similar outcome of inhibition – manifesting in longer reading and response times – has been observed in most studies.

2. For present purposes, we do not distinguish between the two accounts; rather, we focus on their alignment in this case, as we believe that both would make similar predictions regarding NS-NNS differences in PO effects, namely, increased PO-induced interference in NNS. Since differentiating between the two is beyond the scope of this study, the reader is directed to Cunnings (2017, 2022) for a more detailed comparison.

3. Note that the dictionary did not include pronunciations for six of the proper names used in our stimuli.

4. Both participant groups also completed other tasks in between blocks or at the end of the experiment; these were part of an unrelated study (Tsoukala et al., 2025) and are beyond the scope of the present investigation.

5. For winsorisation, we followed Tukey’s rule to identify values that exceeded 1.5 times the interquartile range (IQR) per variable. As such, data points that fell above or below these IQR-based bounds were replaced by the maximum upper or minimum lower bound values, respectively.

6. Because mean-centring eliminates participant-level variance, random intercepts for participants were excluded from these models to avoid singular fit issues.
